# Expression of chemosensory proteins in the tsetse fly *Glossina morsitans morsitans* is related to female host-seeking behaviour

**DOI:** 10.1111/j.1365-2583.2011.01114.x

**Published:** 2011-11-11

**Authors:** R Liu, X He, S Lehane, M Lehane, C Hertz-Fowler, M Berriman, L M Field, J-J Zhou

**Affiliations:** * Department of Biological Chemistry, Rothamsted ResearchHarpenden, UK; † Liverpool School of Tropical MedicinePembroke Place, Liverpool, UK; ‡ Parasite Genomics, Wellcome Trust Sanger InstituteHinxton, UK; § Zhejiang Academy of Agricultural SciencesHangzhou, China

**Keywords:** chemosensory protein, tsetse fly, gene expression, trypanosomiasis, nagana

## Abstract

Chemosensory proteins (CSPs) are a class of soluble proteins present in high concentrations in the sensilla of insect antennae. It has been proposed that they play an important role in insect olfaction by mediating interactions between odorants and odorant receptors. Here we report, for the first time, the presence of five CSP genes in the tsetse fly *Glossina morsitans morsitans*, a major vector transmitting nagana in livestock. Real-time quantitative reverse transcription PCR showed that three of the CSPs are expressed in antennae. One of them, *GmmCSP2*, is transcribed at a very high level and could be involved in olfaction. We also determined expression in the antennae of both males and females at different life stages and with different blood feeding regimes. The transcription of *GmmCSP2* was lower in male antennae than in females, with a sharp increase in 10-week-old flies, 48 h after a bloodmeal. Thus there is a clear relationship between CSP gene transcription and host searching behaviour. Genome annotation and phylogenetic analyses comparing *G. morsitans morsitans* CSPs with those of other Diptera showed rapid evolution after speciation of mosquitoes.

## Introduction

Tsetse flies are a group of insect species that vector trypanosomes, causing sleeping sickness in humans and other animals. One species, *Glossina morsitans morsitans*, has a massive economic impact on African development because of its transmission of nagana in livestock. It is estimated that this disease results in approximately 3 000 000 cattle deaths per year and also limits the supply of meat and milk products and the availability of animal labour for ploughing (Aksoy *et al*., 2005). Tsetse flies are attracted to their hosts by a range of signals including chemical cues, and currently one of the major control methods for tsetse flies is the use of insect traps and insecticide-treated targets, which use both visual and chemical cues to lure the flies to the traps. Repellent chemicals are also used to protect humans and animals from being bitten. Thus there is a need to understand the molecular basis of olfaction in tsetse flies, including odorant binding proteins (OBPs) and chemosensory proteins (CSPs) in the antennae.

CSPs and OBPs are two classes of soluble proteins found in the sensillum lymph of insect antenna. The major difference is that CSPs have a conserved four-cysteine signature (C_1_-X_6_-C_2_-X_6-18_-C_3_-X_2_-C_4_) and OBPs have a conserved six-cysteine signature (C_1_-X_20-35_-C_2_-X_3_-C_3_-X_20-30_-C_4_-X_8-12_-C_5_-X_8_-C_6_) resulting in very different 3D protein structures (Campanacci *et al*., 2001; Mosbah *et al*., 2003; Tomaselli *et al*., 2006). OBPs have been shown to be involved in the first step of olfactory molecular recognition and signal transduction by ferrying airborne host odorants across the sensillum lymph to the odorant receptors. CSPs were first discovered and named as olfactory-specific protein D (OS-D) in *Drosophila melanogaster* by McKenna *et al*. (1994). In fact the CSP domain (pfam03392) used in this work is derived from the conserved sequence alignments profiles based on a collection of OS-D-like CSP sequences and referred to as OS-D domain (http://www.ncbi.nlm.nih.gov/cdd?term=pfam03392). CSPs are secreted into the sensillum lymph of insect chemosensory sensilla and it has been proposed that they are involved in CO_2_ detection, in chemical signal transmission, in regenerating legs and in chemo-perception (olfaction and taste), based on whether they are present in antennae, tarsi or the labarum. Indeed, although many are expressed in the antennae, others are expressed in other tissues including legs (Mameli *et al*., 1996; Picimbon *et al*., 2001), labial palps (Maleszka & Stange, 1997), tarsi (Angeli *et al*., 1999), brain (Whitfield *et al*., 2002), proboscis (Nagnan-Le Meillour *et al*., 2000), pheromone gland (Jacquin-Joly *et al*., 2001; Dani *et al*., 2011) and wings (Ban *et al*., 2003). In *Apis mellifera* CSPs have been reported to be involved in larval development and brood pheromone transportation (Briand *et al*., 2002; Forêt *et al*., 2007). In the cockroach *Blatta germanica* a CSP is involved in leg regeneration (Kitabayashi *et al*., 1998). One CSP of the diamond-back moth, *Plutella xylostella*, is able to bind nonvolatile oviposition deterrents (X. Liu *et al*., 2010). Several CSPs are highly expressed in the lymph of chemosensilla and exhibit binding activity towards odorants and pheromones (Pelosi *et al*., 2006), but there is little evidence of a role in olfaction.

In the present study we constructed and sequenced an antennal cDNA library of *G. m. morsitans* and searched all other available expressed sequence tags (ESTs) and genome shot-gun data. The expression of all of the CSP genes was analysed in heads, bodies and antennae. In order to associate the CSP genes with host location or sex pheromone detection, the transcription profiles of the CSP genes in the antennae were analysed separately in males and females using different starvation regimes. Phylogenetic relationships of the CSPs between *G. m. morsitans* and other Diptera were also used to determine the evolution of CSP genes in Diptera.

## Results and discussion

### Identification of CSPs in *G. m. morsitans*

**Figure 1:**
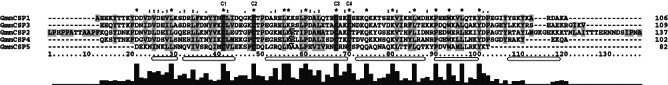
Alignment of chemosensory proteins (CSPs) of *Glossina morsitans morsitans*. The conserved four-cysteine signature of CSPs is shaded in dark grey. The hydrophobic positions in the alignment with the cut-off percentage ≥60% are shaded in light grey. The horizontal bars under the alignment represent the *α*-helices of *Bombyx mori* CSP1 (Jansen *et al*., 2007). The intron splice site was separated by the angle separator. The numbers below the sequences are the residue numbering. Asterisks (*) indicate positions that have a single, fully conserved residue. Colons (:) indicate conservation between groups with very similar properties – scoring >0.5 in the Gonnet PAM 250 matrix. A full stop (.) indicates conservation between groups with slightly similar properties – scoring ≤0.5 in the Gonnet PAM 250 matrix (Larkin *et al*., 2007). These are also indicated by the height of the dark bars in the bottom panel.

Reverse position specific BLAST (RPS-BLAST) was used to search for the OS-D domains (pfam 03392) against all ESTs of *G. m*. *morsitans* in GenBank, including the antennae cDNA library data (R. Liu *et al*., 2010), and the genomic data sequenced (http://ftp://ftp.sanger.ac.uk/pub/pathogens/Glossina/morsitans/). All the sequences with an OS-D domain (with a cut-off threshold of 10^−5^) were deemed to be candidate CSPs, giving 29 ESTs and 28 genomic sequences (from 75 278 ESTs and 1536 Mb of genomic sequences). The candidate genes were assembled into five contigs, all with the typical four-cysteine signature (C_1_-X_6_-C_2_-X_6-18_-C_3_-X_2_-C_4_) of CSPs, showing that *G. m. morsitans* has five independent CSP genes with from 108 to 158 amino acids (Table 1). All five predicted CSPs have a predicted signal peptide, varying from 19 to 26 amino acids and indicating that the CSP sequences are full length. The mature peptides were aligned (Fig. 1) revealing the presence of the expected four cysteines. Besides the four-cysteine signature, some hydrophobic residues are also highly conserved (Fig. 1) and these may be critical for the specific 3D structural configuration of the CSPs (Campanacci *et al*., 2003; Tomaselli *et al*., 2006; Jansen *et al*., 2007; Zhou *et al*., 2009).

**Table 1:** Deduced chemosensory proteins (CSPs) identified from genome and expressed sequence tag (EST) sequences of *Glossina morsitans morsitans*

Name	Length	GenBank ID	Closest CSP* (identities, E-value)	Sources†	Signal peptide
GmmCSP1	126	FN432801	PebIII (69%, 1e-42)	L, A, F, G, R, S, T	1-20aa
GmmCSP2	158	FN432802	a10 (66%, 1e-50)	A, T	1-21aa
GmmCSP3	128	FN432803	PebIII (70%, 3e-45)	A, T	1-19aa
GmmCSP4	123	FN432804	Phk-3 (65%, 9e-46)	T	1-21aa
GmmCSP5	108	FN432805	DmelCSP1 (73%, 2e-43)	T	1-26aa

* The proteins in this column are all CSPs of *Drosophila melanogaster*.

† Sequences were identified from EST libraries of antennae (A), head (H), larvae (L), pupae (P), reproductive organs (R), fat body (F), adult gut (G) and salivary gland (S) as well as genomic trace data (T).

Three of the *G. m. morsitans* CSP genes (*GmmCSP2*, *GmmCSP4* and *GmmCSP5*) have an intron between conserved cysteines C_2_ and C_3_ (Fig. 1). Interestingly, the intron between C_2_ and C_3_ in *GmmCSP4* is lost in the *Drosophila* orthologue Phk-3.

**Figure 2:**
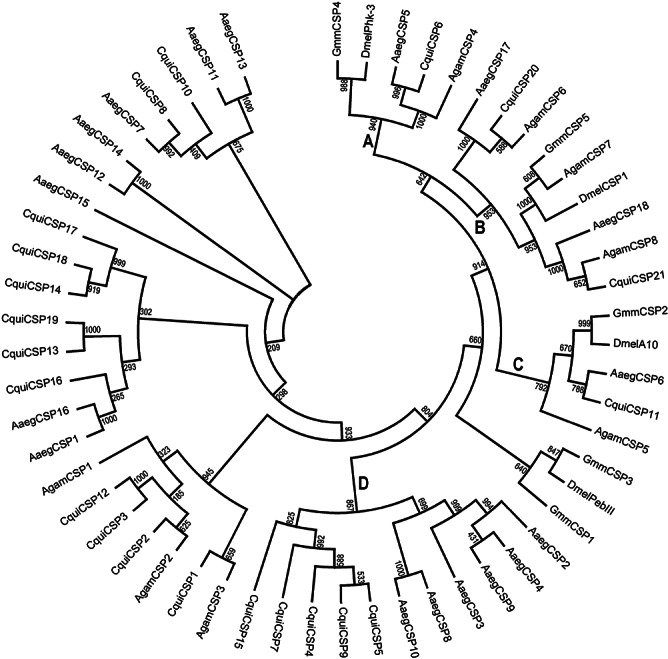
Phylogenetic tree of chemosensory proteins (CSPs) in the dipteran species *Glossina morsitans morsitans* (Gmm), *Drosophila melanogaster* (Dmel), *Aedes aegypti* (Aaeg), *Anopheles gambiae* (Agam) and *Culex quinquefasciatus* (Cqui). Bootstrapping supports are indicated beside the branches at 1000 simulations. Subtrees A to C show consensuses in all the five species. Subtree D shows recent duplication of some CSPs after the divergence of the *Aedes* and *Culex* groups.

Phylogenetic analysis of the five *G. m. morsitans* CSPs (Fig. 2) shows that they are very diverse as seen in CSPs of other insect species (Pelosi *et al*., 2006; Xu *et al*., 2009). Only two CSPs (GmmCSP1 and GmmCSP3) are clustered together with bootstrapping support of 70%. Their amino acid sequences are 63% identical and are orthologues of the *Drosophila* CSP PebIII. The closest orthologues to the *G. m. morsitans* CSPs are found in *D. melanogaster* with an average protein sequence identity of 69% (Table 1). ESTs of *GmmCSP1* were found from cDNA libraries of larvae, antennae, fat body, adult gut, reproductive organs and salivary gland, whereas ESTs of *GmmCSP3* were only present in the antennal library. This indicates that *GmmCSP1* may have a range of functions, depending on where it is expressed, but *GmmCSP3* may be involved in olfaction (see below), with the two genes gaining different functions during their divergence from the ancestor gene.

### Tissue distribution of CSP genes

**Figure 3:**
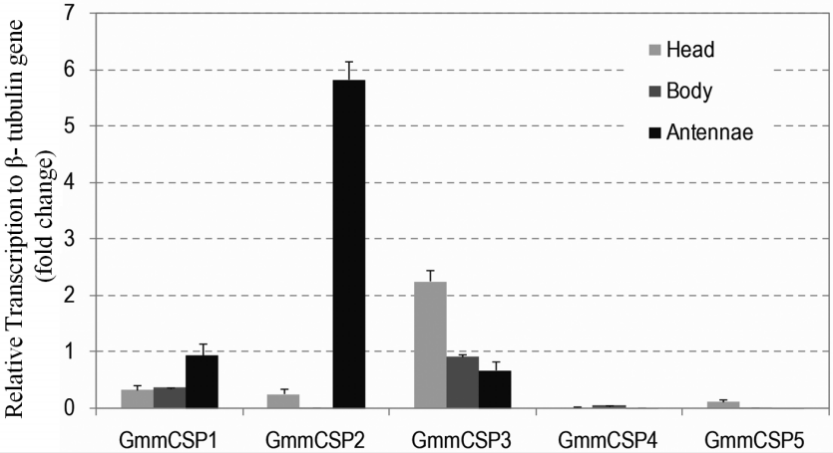
Transcript abundance of cDNAs encoding chemosensory proteins (CSPs) in *Glossina morsitans morsitans* as determined by quantitative reverse transcription PCR (qPCR). The mean values and standard errors of four replicates are presented relative to the internal control gene. Two qPCRs were performed for each of two RNA preparations from each tissue sample.

In order to examine further the potential functions of the *G. m. morsitans* CSPs and which CSP gene is expressed in the olfaction organs of *G. m. morsitans*, we carried out a real time quantitative reverse transcription PCR (qPCR) analysis to measure the transcription abundance of the CSP genes in head plus antennae, body and antennae alone (Fig. 3). Of the five CSP genes, two (*GmmCSP1* and *GmmCSP2*) had higher transcription levels in antennae, with *GmmCSP2* being about five times higher than in other tissues. Furthermore, expression of *GmmCSP2* was not detected in bodies, and was very low in heads plus antennae (about 0.26 times that of *β-tubulin*) probably because of the presence of antennal tissues, indicating that *GmmCSP2* is specifically, and highly, expressed in the antennae and could therefore play a role in olfaction. Transcription of *GmmCSP1* and *GmmCSP3* was detected in all the tissues tested, with *GmmCSP1* having a higher transcription level in antennae relative to heads and bodies, about 0.94, 0.34 and 0.36 times higher than that of *β-tubulin*, respectively. *GmmCSP3* was more highly transcribed in heads than in bodies and antennae, about 2.3, 0.92 and 0.68 times that of *β-tubulin*, respectively. The higher transcription in heads suggests that *GmmCSP3* may have other possible roles apart from olfaction. Our qPCR analysis provided the expression profile information of CSP genes in the heads and bodies that was lacking in the EST data (Table 1). *GmmCSP4* and *GmmCSP5* were only weakly transcribed in all adult tissues and may not play any olfactory roles in adult tsetse flies.

### Expression of *G. m. morsitans* CSPs following a bloodmeal

**Figure 4:**
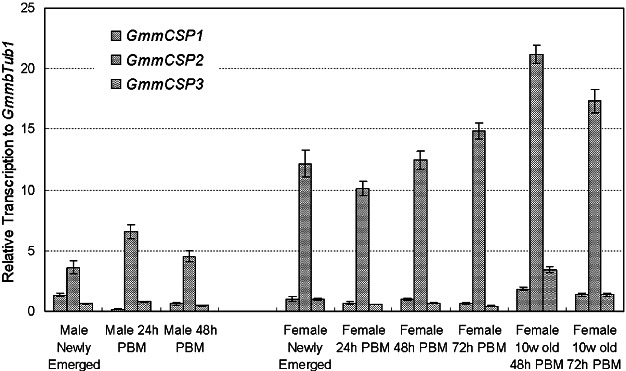
Transcription of chemosensory protein genes in the antennae of *Glossina morsitans morsitans*. The transcription levels are presented as mean fold changes relative to the internal reference with four replicates. PBM, postbloodmeal; w, week. Two quantitative reverse transcription PCRs were performed for each of two RNA preparations from each tissue sample.

To characterize further the CSP genes in the antennae of *G. m. morsitans*, we dissected antennae tissues from male and female flies before and after a bloodmeal at a range of time intervals (Fig. 4). For males the antennae were collected from newly emerged adults, 24 and 48 h postbloodmeal (PBM). For young females the antennae were further collected 72 h PBM, and for 10-week-old females the antennae were collected 48 and 72 h PBM. Of the three CSP genes transcribed in antennae (Fig. 3), *GmmCSP1* and *GmmCSP3* had similar transcription levels in males and females and there were no notable differences before and after a bloodmeal up to 48 h. However, *GmmCSP2* had higher transcription in females than in males, being 3.3, 1.5 and 2.5 times higher in female antennae than in male antennae for newly emerged, 24 h and 48 h PBM adults, respectively (Fig. 4). In the newly emerged flies the transcription of *GmmCSP2* was 12.2 ± 1.1-fold higher than that of *β*-*tubulin* in female antennae and 3.6 ± 0.5-fold higher than *β*-*tubulin* in male antennae. Moreover, in females *GmmCSP2* transcription was decreased within 24 h PBM and then increased with time from 10.1 ± 0.6-fold higher than *β*-*tubulin* transcription at 24 h PBM to 12.5 ± 0.8 and 14.9 ± 0.7-fold higher at 48 and 72 h PBM, with a further increase to 21.2 ± 0.7-fold higher in 10-week-old females. This increase may be related to the higher demand for food during starvation and the need for an increased ability to detect hosts by the female flies. Besides the relationship with starvation, *GmmCSP2* transcription level was also related to age, with older flies having a higher transcription level than with young flies at similar stages of starvation (48 h PBM).

### Evolution of CSPs in dipteran insects

To characterize the molecular evolution relationships between the CSPs of *G. m. morsitans* and those of others insects in Diptera, we constructed a phylogenetic tree (Fig. 2) including five CSPs of *G. m. morsitans* identified in this study, 21 in *Culex quinquefasciatus* (Pelletier & Leal, 2011), four in *D. melanogaster* (McKenna *et al*., 1994; Zhou *et al*., 2006), eight in *Anopheles gambiae* (Zhou *et al*., 2006) and 18 in *Aedes aegypti* (J.J. Zhou, unpubl. data). The CSP sequences are listed in Table S2. The tree shows that each *G. m. morsitans* CSP has orthologues in *D. melanogaster* with strong bootstrapping support: GmmCSP1 and DmelPebIII, GmmCSP3 and DmelPebIII, GmmCSP2 and DmelA10, GmmCSP4 and DmelPhk-3, GmmCSP5 and DmelCSP1, consistent with the established species phylogeny that *G. m. morsitans* is more closely related to *D. melanogaster* than to mosquitoes (Arensburger *et al*, 2010). Interestingly, the antenna-specific CSP of *G. m. morstans* GmmCSP2 is the orthologue of DmelA10 or OS-D, a CSP from *D. melanogaster*. DmelA10 protein was found to express in the sensillum coeloconicum of antennal segment 3, which is the main olfaction organ of *D. melanogaster* (McKenna *et al*., 1994; Pikielny *et al*., 1994). *GmmCSP2* was also found to be more highly expressed in female than in male tsetse flies (Fig. 4). *GmmCSP1* and *GmmCSP3* are expressed in heads, bodies and antennae and are orthologous to DmelPebIII (McKenna *et al*., 1994). *DmelPebIII* expression was found to be induced by viral and bacterial infections (Sabatier *et al*., 2003) and expressed at a high level in antennae in adult heads, eyes, crops, ejaculatory bulbs and hindguts [FlyBase: Gelbart, W.M., Emmert, D.B. (2010.10.13) FlyBase High Throughput Expression Pattern Data Beta Version], consistent with the nonspecific expression profile of *GmmCSP1* and *GmmCSP3* (Fig. 3).

The tree also shows three orthologous groups containing CSP members from all five species, labelled as A, B and C in Fig. 2. These appear to represent three lineages in Diptera that diverged about 210–260 million years ago (Ma), before the divergence of Brachycera and Nematocera (Arensburger *et al*, 2010). GmmCSP1, GmmCSP3 and DmelPebIII are clustered together in a separate branch and may have diverged after the split of Brachycera (flies) and Nematocera (mosquitoes). A gene expansion branch, labelled D in Fig. 2 shows that these CSPs diverged after the split of *Aedes* and *Culex*, and indicates a rapid CSP gene expansion around 50–54 Ma in both mosquito species.

There seems to be a correlation of the expansion of CSP genes and the number of CSPs in dipteran species. *Aedes aegypti* with 18 CSPs and *Culex quinquefasciatus* with 21 CSPs diverged from Culicinae about 50–54 Ma, whereas *G. m. morsitans* with only five CSPs and *D. melanogaster* with only four CSPs diverged from Brachycera about 210–260 Ma. *Anopheles gambiae*, with an intermediate number of eight CSPs, diverged from Culicinae about 150 Ma (ie between the 50–54 and the 210–260 Ma period of the divergence of Brachycera and Nematocera). Thus, dipteran CSPs may have evolved from three ancestral CSP genes about 260 Ma, during the diversification of suborders Nematocera and Brachycera. The expansion of CSPs then slowed down in insect species of the Brachycera, but continued in insect species of the Nematocera about 150 Ma, with a rapid expansion in insect species of the Culicinae in the last 50–54 million years.

## Experimental procedures

### Insects and tissues

The *G. m. morsitans* colony was maintained at the Liverpool School of Tropical Medicine (colony established in 2002 from the Bristol colony, itself originally derived from flies from Zimbabwe). Flies were kept at 26 °C and 70% relative humidity. Male flies were fed with defibrinated horse blood every 48 h through artificial membranes (Moloo, 1971). Twenty-four hours after a fresh bloodmeal, flies were frozen at −20 °C for 5 min, and then the heads with antennae and bodies were separated and placed in 100 µl ice-cold Trizol regent (Invitrogen, Paisley, UK) for RNA extractions.

Antennae were prepared from (1) newly emerged males, (2) young males 24 h PBM, (3) young males 48 h PBM, (4) newly emerged females, (5) young females 24 h PBM, (6) young females 48 h PBM, (7) young females 72 h PBM, (8) 10-week-old females 48 h after last bloodmeal and (9) 10-week-old female 72 h after last bloodmeal. Living flies were chilled at 4 °C then antennae were detached (using sterile fine forceps) and immediately placed in an Eppendorf tube containing 100 µl ice-cold Trizol reagent. About 50–60 pairs of antennae were collected from each sample.

### RNA and DNA preparation

Twenty heads with antennae weighing *c*. 42 mg and four bodies without heads weighing *c.* 92 mg were ground separately in liquid nitrogen. The powder was then mixed with Trizol regent (1 ml) and ground again. The homogenized tissue was then transferred into a 1.5-mL RNase-free tube, mixed with 200 µl chloroform and left for 10 min at room temperature. For antennal RNA isolation, about 60 pairs of antennae were ground directly in a 1.5 ml Eppendorf tube with 500 µl Trizol reagent using a plastic pestle. Total RNA was extracted using Trizol reagent according to the manufacturer's instructions (Invitrogen). Two biological replicates of RNA were prepared from each antennal sample.

Genomic DNA was isolated to make standard curves for qPCR. The body tissues of a tsetse fly without the head were homogenized in a 1.5-mL Eppendorf tube with 250 µl DNA extraction buffer (100 mM Tris-HCl pH 9.0, 100 mM ethylenediaminetetraacetic acid, 1% sodium dodecyl sulphate). The mixture was then heated at 70 °C for 30 min, mixed with 35 µl of 8 M potassium acetate solution and incubated on ice for 30 min. The supernatant containing DNA was obtained by centrifugation of the extraction mixture at 12 054 ***g*** for 10 min, and then extracted further with 280 µl chloroform : phenol 1:1. The DNA sample was treated with 2 µl RNase (10 mg/ml) at 37 °C for 15 min, extracted again with 250 µl chloroform, and finally DNA was precipitated with 2.5 volume of 100% ethanol. The DNA pellet was washed with 75% ethanol and dissolved in 60 µl water (Sigma, St Louis, MO, USA). Genomic DNA samples of 20, 2, 1 and 0.2 ng/µl were used to plot standard curves for calculating the transcript abundance of each gene.

### Construction of the antennal cDNA library

The antennal cDNA library was constructed using the Creator Smart cDNA Library Construction Kit (Clontech, Mountain View, CA, USA) according to the supplier's instructions. About 700 ng antennal RNA from flies of mixed ages and sexes was used for the first strand cDNA synthesis in a reaction volume of 10 µl, from which 2 µl single strand cDNA was used for long distance PCR with pre-denaturing at 95 °C for 2 min, followed by 20 cycles of 95 °C for 15 s and 68 °C for 6 min and a final elongation cycle of 72 °C for 2 min. The purified resultant double strand cDNA was digested with *Sfi*I, size fractionated and ligated into the pDNR-Lib vector (Clontech). The ligation mixture was desalted, electroporated into *Escherichia coli* XL1-blue electro-competent cells and plated on agar plates supplemented with chloramphenicol (34 µg/ml). Clones were placed into the wells of 384-well plates for sequencing of randomly selected bacterial clones in both directions using T3 and T7 primers and ABI Big Dye Terminator Cycle Sequencing kits (Life Technologies Ltd, Paisley, UK). The raw sequences were clipped in Phred to remove the unqualified ends and the vector sequences were removed with Cross Match software (P. Green, unpublished). Sequence reads were assembled into clusters with Phrap (P. Green, unpublished; http://www.phrap.org).

### Identification of putative CSP cDNAs in the *G. m. morsitans* library

Sequences from the antennal cDNA library (this study) and of other ESTs (http://www.ncbi.nlm.nih.gov/dbEST/) as well as the whole-genome shotgun reads and the genome assembly produced by the Wellcome Trust Sanger Institute (available from http://ftp://ftp.sanger.ac.uk/pub/pathogens/Glossina/morsitans/) were searched with a combination of methods using MotifSearch (Zhou *et al*., 2004, 2008), BLASTx, on the National Center for Biotechnology Information (NCBI) website (http://blast.ncbi.nlm.nih.gov/Blast.cgi) and RPS-BLAST (Marchler-Bauer *et al*., 2011). The sequence hits were collected as putative CSP sequences if their BLAST scores were less than 10^−5^ compared to the OS-D domain (pfam03392) and to known CSP sequences and they had the CSP four cysteine signature, with a low molecular weight and a hydrophobic signal peptide.

### qPCR

Primers were between 19 and 22 bp with the melting temperature (*T*_m_) values ranging from 59.5 to 60.5 °C and designed using Primer3 Plus (http://www.bioinformatics.nl/cgi-bin/primer3plus/primer3plus.cgi). The size of the PCR products was set within the range 120–260 bp. As genomic DNA was used to establish standard curves for quantification, the primers were optimized and designed on single exon or exons flanking short introns. Information on the primers is given in Table S1.

Invitrogen Platinum SYBR Green qPCR SuperMix-uracil DNA glycosylase (Invitrogen) was used for the qPCR reactions. An aliquot (5 µl) of total RNA ranging from 0.2 to 1 µg was first treated with RQ1 DNase in a total volume of 10 µl reaction mixture. Then, 6 µl reaction mixture was used for reverse transcription using the ImProm-II Reverse Transcription System (Promega, Southampton, UK) in 20 µl reactions and then diluted to 500 µl, from which 3 µl was used as the template for each qPCR reaction. Each reaction included 1 × SuperMix, 200 nM of each of the gene-specific primer pairs, 50 nM 6-carboxy-X-rhodamine dye and 3 µl templates. The qPCRs were carried out for each of two RNA preparations from each tissue sample. The *β-tubulin* gene of *G. m. morsitans* was included for initial normalization of the template amount. The PCR reactions were carried out using a Stratagene Mx3000P qPCR system (Agilent Technologies UK Ltd, Cheshire, UK) with a thermoprofile of one cycle of 50 °C for 2 min, 95 °C for 2 min, then 45 cycles of 95 °C for 15 s, 60 °C for 45 s, followed by a melting curve analysis from 55 to 95 °C.

## Supporting Information

**Table S1.** Primers used for real timequantitative reverse transcription PCR analysis of chemosensoryprotein genes in *Glossina morsitans morsitans*.

**Table S2.** The GenBank ID, name and amino acid sequence of chemosensory proteins that are used to construct the phylogenetic tree (Fig. 2).

Please note: Neither the Editors nor Wiley-Blackwell are responsible for the content or functionality of any supporting materials supplied by the authors. Any queries (other than missing material) should be directed to the corresponding author for the article.
