# Supplementary material for: Expression of chemosensory proteins in the tsetse fly Glossina morsitans morsitans is related to female host-seeking behaviour
Source: Insect Mol Biol. 2011 Nov 11;21(1):41–8. doi: 10.1111/j.1365-2583.2011.01114.x (PMC3664020; doi:10.1111/j.1365-2583.2011.01114.x)
Supplement: Supplementary file 1 [file imb0021-0041-SD1.doc]

***Supplementary Table 1.*** *Primers used for qRT-PCR analysis of CSP genes in* Glossina morsitans morsitans

| Gene | Forward primer | Reverse primer | Tm (℃)  Fwd;Rev | Amplicon (bp)  cDNA;gDNA |
| --- | --- | --- | --- | --- |
| *GmmCSP1* | atgacgtcgatgtcgatgag | gcaagaccttccattcttcg | 59.7;59.8 | 228;228 |
| *GmmCSP2* | tacggttcggagaaggtgac | tttaccatcgacgtcaagca | 60.1;60.3 | 202;202 |
| *GmmCSP3* | cgttgtcgctgttgtcctta | accttcgggtgtgcatttac | 59.9;59.9 | 160;160 |
| *GmmCSP4* | acgcgttgatatcggactgt | ccggaaggatcgtatttattca | 60.5;60.2 | 130;130 |
| *GmmCSP5* | tgctttacccgaggttatcg | cttaggagcatcgcccatac | 60.1;59.7 | 123;123 |
| *GmmAct1* | cgaagaacatcccgtcttgt | gaggatagcgtgaggcaaag | 60.1;60.0 | 235;235 |
| *GmmβTub1* | gtttgaaaatgtcggccact | tcctcttcaaattcggcatc | 60.0;60.2 | 238;238 |
